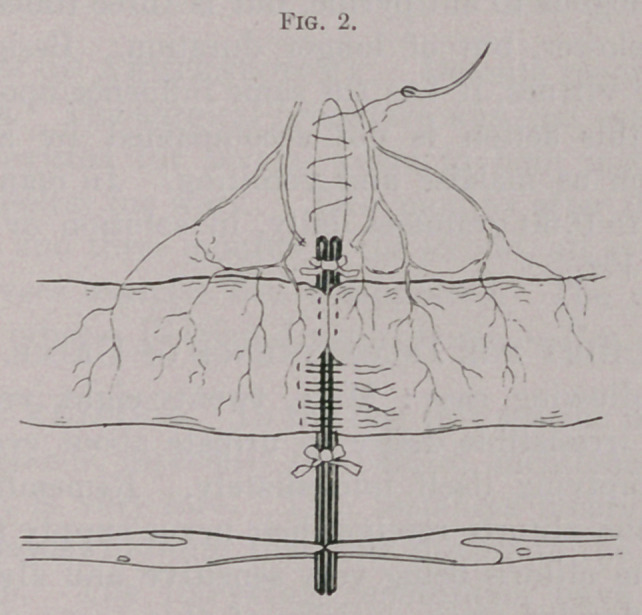# Department of Canine, Feline and Avian Medicine and Surgery

**Published:** 1902-03

**Authors:** Cecil French

**Affiliations:** D.V.S. (McGill and Munich), Washington, D. C.


### DEPARTMENT OF CANINE, FELINE, AND AVIAN MEDICINE AND SURGERY.

By Cecil French, D.V.S. (McGill and Munich),
WASHINGTON, D. C.

### A SIMPLE METHOD OF PERFORMING ENTERO-ENTERAL ANASTOMOSIS IN THE DOG.

Resection of the bowel is an operation the veterinarian is rarely called upon to perform, but it becomes necessary when a portion of the intestine has lost its viability. Such condition arises most commonly consequent upon acute intestinal obstruction or strangulation.

Removal of more than one-third the length of the small bowel is dangerous to life. Parkes (*Gunshot Wounds of the Small Intestines*) found that recovery occurred most readily when the portion of bowel resected did not much exceed six inches. Experiments demonstrated that extensive resection, where the resected portion exceeded one-half the length of the intestinal tract and where the animals survived the operation, was followed by marasmus as a constant result, though the animals consumed large quantities of food.

Any method of performing this operation demands great precision and attention to detail, but if undertaken in good time offers reasonable hope of success.

A great many devices have been contrived to facilitate anastomosis. Some of them are merely intended to assist in holding the cut ends of the bowel in apposition while sutures are being applied, and take no further part in effecting the reunion. Others allow the operator to dispense with all or most of the suturing, but must necessarily remain in position, holding the cut ends in apposition sufficiently long for reunion to be established. Some of the latter —particularly those which are unabsorbable, like the Murphy button—hold the ends together by compression, thus producing more or less local gangrene, and finally slough away and are voided. This feature of pressure-gangrene production constitutes a pronounced defect, and all mechanical devices which depend upon it for the desired effect are necessarily active irritants and a menace to the reparative capacity of the parts. In fact, the best method must be that which dispenses altogether with the presence of any foreign body, except it be to lend temporary support to the parts during suturing.

But without the employment of some kind of supporting device the operation is rendered vastly more difficult. When the intestine of the dog is severed the muscular coat immediately begins to contract. The diameter of the tube is often diminished more than one-half. The circular layer, which is thickest, causes the sudden narrowing of the lumen, and the longitudinal layer then coming into play brings about a pronounced eversion of the mucous membrane. This action can be overcome to a considerable degree by gently inserting the tip of the little finger within the lumen of the severed ends, but even then it is a matter of extreme difficulty to maintain the cut ends in apposition while the suturing is being carried out.

For the veterinary practitioner a method is needed which does not involve the employment of specially manufactured devices, which, excepting in large cities, are unobtainable at short notice. The necessity for performing the operation invariably arises as an emergency ; hence it is indispensable that the technique be as simple as is compatible with favorable results, and that any device necessary to facilitate the work be such as may be fashioned out of material at hand and at short notice. With this end in view I have contrived a method which I believe to be peculiarly adapted to canine practice. The sole device of which it is necessary to make use is a lady's hairpin, bent as figured in the accompanying illustrations. Two of these are required, together with three or four pairs of hæmostatic forceps to act as clamps.

For the suturing a milliner's needle, size 8 or 9, should be employed, and the suturing material should be the finest No. 2 black sewing silk. Nearly every surgeon of note who has experimented on dogs recommends silk. Thick catgut remains unchanged not over seven days as a rule, which is not a period of sufficient duration for certain coalescence to take place, and when tied the knots interfere with accurate approximation. Fine catgut disappears in less time, while aseptic silk threads can be tied with greater accuracy, and the knots never become loosened, and its permanent presence never exerts any ill-effect, for it becomes solidly encapsulated. In place of silk, I have used ordinary sewing cotton with good results.

In order to follow each successive step in a complete enterectomy and end-to-end anastomosis by this method, let us suppose that on exposing the viscera a tract of small intestine is found to be in an advanced state of gangrene from the presence of some obstruction or owing to strangulation. The operator must first carefully inspect the local blood-supply, bearing in mind that no mesenteric vessels must be obliterated other than those supplying the area of intestine it is intended to remove. This precaution must be rigidly observed, because it is of the utmost importance that the circulation be preserved up to the very row of sutures. All the circulation possible is needed to effect rapid coalescence of the parts and to ward off further gangrene. The area of intended resection and the blood-supply of the same being mapped out, the mesenteric vessels are first secured by ligature, which is best done by means of a curved needle and fine silk suture passed through the mesen-

Fig. 1.

tery and around them. The anastomosing loops running near the mesenteric attachment are secured at a point level with the proposed line of resection.

One prong of the hairpin is passed through the mesentery at the upper point of resection, and, together with its fellow, is brought transversely across the gut. The two are clamped together with hæmostatic forceps. The other pin is affixed in the same manner at the lower line of resection. No other bowel clamp is needed, as the lumen is closed from the outset.

The intestine is severed with the scalpel quite close to the clamped prongs of the pin, and the incision extended to the mesentery so as to remove a wedge-shaped portion. The pin effectually inhibits all vermicular action of the wall. The two pins are approximated and tied tightly together, or they may be locked by means of additional hæmostatic forceps. The sutures are now placed on one side, starting at the mesenteric attachment. They are tied before proceeding to the other side. The bowel is turned over and the sutures are applied in the same manner on the other side. The pins are then untied and unclamped and severed at their bent ends with bone forceps or stout scissors, and withdrawn, one

FIG. 2.

prong at a time. The remaining openings are closed with one stitch each, particular care being exercised that the margin is properly turned in at the mesenteric attachment. Finally, the incision in the mesentery is closed with a continuous suture.

I have performed this operation experimentally on six different animals, all of which recovered except one, which was only five weeks old, and which survived from the actual operation, but succumbed later to obstruction of the canal owing to the formation of adhesions between the line of coalescence and one side of the wall immediately beyond, whereby an acute flexure was developed.

## Figures and Tables

**Fig. 1. f1:**
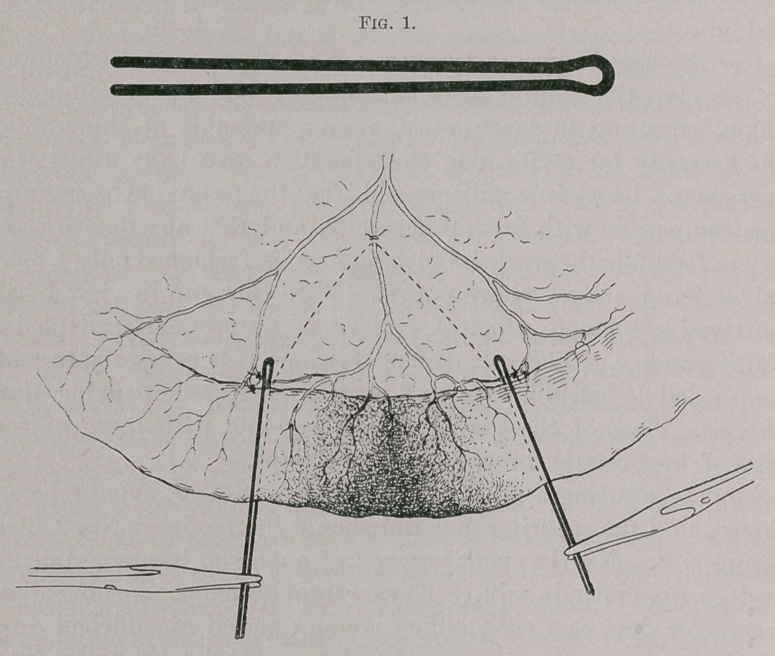


**Fig. 2. f2:**